# 3-HK SUPPLEMENTATION INCREASES NICOTINATE PHOSPHORIBOSYLTRANSFERASE (NAPRT) EXPRESSION IN DROSOPHILA

**DOI:** 10.1093/geroni/igad104.3137

**Published:** 2023-12-21

**Authors:** Qiao Huang, Adam Said, Yuqiong Wu, Jeremy Walston, Reyhan Westbrook, Peter M Abadir

**Affiliations:** Johns Hopkins University School of Medicine, Baltimore, Maryland, United States; Emory College of Arts and Sciences, Atlanta, Georgia, United States; Johns Hopkins University School of Medicine, Baltimore, Maryland, United States; Johns Hopkins University, Baltimore, Maryland, United States; Johns Hopkins University School of Medicine, Baltimore, Maryland, United States; Johns Hopkins University School of Medicine, Baltimore, Maryland, United States

## Abstract

The increasing prevalence of physical frailty and aging related diseases in older adults necessitates the development of novel biological strategies to slow age-related declines. In humans, tryptophan metabolism through the kynurenine pathway (KP), which produces the energy metabolite and cellular cofactor nicotinamide adenine dinucleotide (NAD+), has been linked to various aging-related diseases including metabolic syndrome, neurodegenerative diseases, and physical frailty. Elevated levels of KP metabolites, such as 3-hydroxykinurenine (3-HK), correlate negatively with lifespan and physical function. A profile of downstream KP metabolites in the serum of older adults showed that 3-HK was increased in frail compared to both non-frail and young subjects, and liver kynurenine/tryptophan ratio correlates negatively with maximum lifespan in twenty-six mammalian species. To comprehensively explore the effects of elevated cytotoxic KP metabolites, we fed DGRP_229 Drosophila males diet infused with 3-HK. We found that treatment with 3-HK reduced average lifespan by 17% (P< 0.0001) compared against control. To elucidate the biological mechanisms of 3-HK treatment, we performed qRT-PCR on 11 genes involved in tryptophan metabolism. We found that treatment with 3-HK significantly decreased the expression of nicotinate phosphoribosyltransferase (Naprt) (P=0.0022), which plays an important role in NAD+ biosynthesis and protects against oxidative stress. Our findings indicate that some of the detrimental effects observed after 3-HK supplementation may be mediated by changes in NAD+ biosynthesis and highlight the potential feasibility of using NAD+ supplementation as an intervention method.

